# Characterization of a model to induce hyperlipidemia in feed-restricted dairy cows

**DOI:** 10.3168/jdsc.2023-0396

**Published:** 2023-08-19

**Authors:** U. Arshad, J.E.P. Santos

**Affiliations:** Department of Animal Sciences, DH Barron Reproductive and Perinatal Biology Research Program, University of Florida, Gainesville, FL 32611

## Abstract

Hepatic lipidosis is a prevalent metabolic disorder, and in vivo models to study intermediary lipid metabolism are needed in dairy cows. Objectives were to apply a method to induce hyperlipidemia and characterize the responses and safety of the intervention in feed-restricted dry Holstein cows at 8 mo of gestation. It was hypothesized that infusion of tyloxapol would induce hyperlipidemia without deleterious effects on health of dairy cows. Pregnant, nonlactating parous Holstein cows (n = 33) at a mean (± standard deviation) of 234 ± 2.2 d of gestation were fed for ad libitum intake on d 1 to 5 and restricted to 41% of the required NE_L_ from d 6 to 13. On d 14, when cows were 247 ± 2.2 d of gestation, cows were kept off feed, and received i.v. a 10% solution of tyloxapol at 120 mg/kg body weight to block hydrolysis of triacylglycerols in very-low-density lipoprotein (VLDL) particles. Blood was sampled for 720 min and analyzed for concentrations of triacylglycerol, VLDL cholesterol, and total cholesterol in serum to reflect hepatic secretion or reduced clearance of such metabolites from blood. Rectal temperature, respiration and heart rates, and clinical signs related to potential anaphylaxis were monitored for the first 30 min relative to tyloxapol infusion, and for any abnormal behavior in the subsequent 24 h. Infusion of tyloxapol progressively increased the concentrations of triacylglycerol, VLDL cholesterol, and total cholesterol in serum. Tyloxapol increased rectal temperature by 0.19°C at 30 min after infusion and increased respiration and heart rates in the first 10 min after infusion by 29% and 40%, respectively. Tyloxapol induced tachycardia (heart rate >80 beats/min) in 66.7% (n = 22), frothy salivation in 39.4% (n = 13), muzzle twitching in 15.2% (n = 5), eyes twitching in 12.1% (n = 4), muscle twitching in 48.5% (n = 16), nystagmus in 6.1% (n = 2), signs of hyperexcitement in 18.2% (n = 6), staggering gait in 18.2% (n = 6), and anaphylaxis in 12.1% (n = 4) of the cows; however, all these signs were transient, and cows returned to normal after 20 min of infusion. No other abnormal behavior was observed past 20 min of tyloxapol infusion. None of the cows aborted and gestation length, calf birth weight, and risk of diseases in the first 21 d postpartum did not differ between cows receiving tyloxapol and a companion group that did not receive tyloxapol. Infusion of tyloxapol induced hyperlipidemia in cows with some animals showing transient reactions to the treatment, but without complications to the cow and the offspring. Application of this model can be useful to study intermediary lipid metabolism in dairy cows.

## Highlights

• Infusion of tyloxapol induced hypertriglyceridemia and hypercholesteremia in cows.
• Some cows showed a transient reaction to the tyloxapol infusion that lasted 20 minutes.
• Infusion of tyloxapol was not associated with effects on the dam or offspring.
• This model can be used to study intermediary lipid metabolism in dry dairy cows.

## Graphical Abstract

**Figure:**
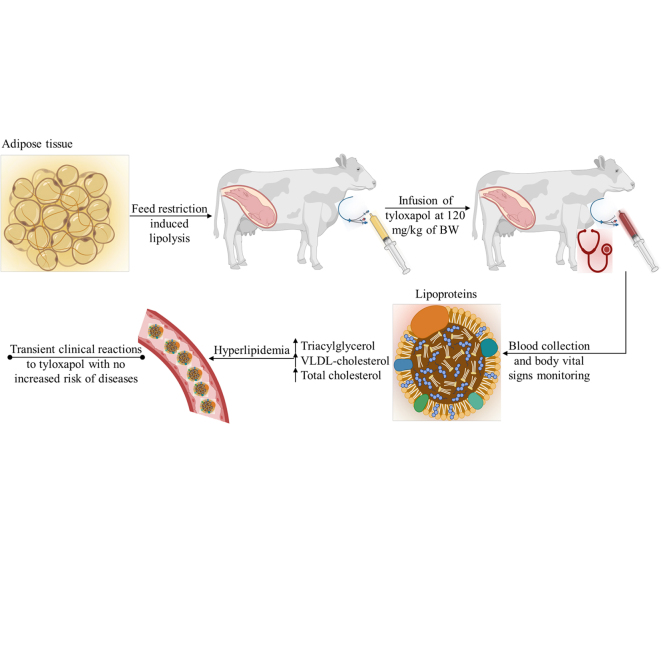
**Summary:** The objectives of this study were to induce hyperlipidemia and characterize the responses and safety of the intervention in dry Holstein cows at 8 months of gestation. Cows were fed for ad libitum intake from day 1 to 5 and restricted to 41% of the net energy for lactation required from day 6 to 13. On day 14, cows were off feed and received an i.v. of solution of 10% tyloxapol at 120 mg/kg body weight to block hydrolysis of triacylglycerols in lipoprotein particles. Blood was sampled for 720 minutes, and vital signs were monitored for 30 minutes. Tyloxapol infusion progressively increased the concentrations of lipids in serum. Some cows showed a transient reaction to the treatment that lasted 20 minutes, but treatment was not associated with negative consequences to health of cows and the offspring. This model can be used to study intermediary lipid metabolism in dry late-gestation dairy cows.

Dairy cows experience increased accumulation of triacylglycerol in the first week after calving (Jorritsma et al., 2001), and the ruminant liver is known to have reduced hepatic secretion of very-low-density lipoproteins (**VLDL**; Emery et al., 1992). Therefore, in vivo models to study intermediary lipid metabolism are needed in dairy cows. It has been shown that acute increases in the blood concentrations of fatty acids result in hepatic uptake of those lipids and re-esterification to triacylglycerol, which causes deposition of lipid droplets into the hepatic parenchyma (Bobe et al., 2004). Depletion of triacylglycerol from bovine liver is a slow process (Bollatti et al., 2020), in part because of the limited export of triacylglycerol as VLDL (Emery et al., 1992). One of the challenges to quantify hepatic export of VLDL in vivo is that it would require measurements of hepatic flux with catheterization of portal and hepatic veins. An alternative that has been used in other animal models is to block catabolism of VLDL and indirectly assess secretion rate by accumulation of triacylglycerol in blood (Fernandez et al., 1997). Triton WR-1339 is a nonionic detergent that blocks the action of lipoprotein lipase in vivo, thus preventing lipolysis of triacylglycerols in lipoprotein particles (Schotz et al., 1957).

Triton WR-1339, also known as tyloxapol, has been used in guinea pigs (Fernandez et al., 1997) and mice (Rinella et al., 2008) to induce hyperlipidemia and quantify triacylglycerol accumulation over time as a measure of hepatic secretion of VLDL; however, limited information is available on the characterization of hyperlipidemia and safety of tyloxapol administration in pregnant cows (Wrenn et al., 1971). We hypothesized that infusion of tyloxapol would induce hyperlipidemia without deleterious effects on the health of dairy cows. Therefore, the objectives of this study were to characterize hyperlipidemia and evaluate vital signs after infusion of tyloxapol in feed-restricted dry dairy cows at mean (± SD) 234 ± 2.2 d of gestation. An additional objective was to evaluate the associations between tyloxapol infusion and gestation length, calf birth weight, and health of dairy cows in the first 21 d postpartum.

The study was conducted at University of Florida Dairy Unit (Hague, FL) and approved by the Institutional Animal Care and Use Committee of the University of Florida (protocol no. 201910612). The design of the present study had 2 components. The first was a longitudinal study to characterize the responses to infusion of tyloxapol in dairy cows. The second component was a cohort study in which cows that received tyloxapol were compared with a cohort that were managed similarly but did not receive tyloxapol.

Pregnant, nonlactating Holstein cows (n = 33) with a mean (± SD) 234 ± 2.2 d of gestation, BCS 3.79 ± 0.49, and 728 ± 72 kg of BW were enrolled in the study. Every 14 d, a cohort of cows was assigned to study. There were 6 cohorts; 5 of them had 6 cows and 1 had 3 cows. For each cow, the study lasted 14 d, followed by a period of observation until the first 21 d of the subsequent lactation. During the 14-d study, cows were fed for ad libitum intake for 5 d and then subjected to feed restriction from d 6 to 13, and then kept off feed on d 14. During the first 5 d of the study, cows were fed a diet containing 1.62 Mcal/kg NE_L_ (NASEM, 2021) and the mean DMI was 8.3 kg/d. During the feed restriction period, the amount of DM offered supplied 41% of the calculated NE_L_ required to meet the needs of maintenance and pregnancy (NASEM, 2021), as part of a protocol to induce hepatic steatosis (Arshad et al., 2023).

On d 13, an i.v. 14-gauge and 9-cm long catheter (Milacath Extended Use, Mila International Inc., Florence, KY) was fit into the jugular vein of each cow and patency was maintained by flushing with 15 mL of sterile heparinized saline containing 10 IU of heparin/mL. Starting at 1400 h of d 13, any feed left in the bunk was removed and cows remained off feed until 2100 h of d 14, with only access to water. On d 14, all 33 cows received an i.v. solution of 10% tyloxapol (product T8761, Sigma-Aldrich, St. Louis, MO), a 4-(1,1,3,3-tetramethylbutyl) phenol polymer with formaldehyde and oxirane, at a dose of 120 mg/kg BW. The weight of cows was calculated as BW minus 50 kg, with the latter representing the expected pregnant uterus weight at 230 d of gestation in a Holstein cow (Bell et al., 1995). Cows remained off feed throughout the 12-h blood sampling after tyloxapol dosing to ensure that the measured serum triacylglycerol concentrations reflected hepatic secretion of VLDL triacylglycerols and not influx of dietary triacylglycerol as chylomicrons. One milliliter of tyloxapol product contained 1.25 g of the active compound, and a 10% (vol/vol) tyloxapol solution was prepared by dissolving and gently mixing the calculated amount of tyloxapol product of each cow in 0.9% sodium chloride solution for 2 h for proper homogenization. The mean (± SD) amount per cow administered was 78.5 ± 8.5 g of the active compound in tyloxapol in a total volume of 628 ± 68 mL, and the duration of infusion was 9.21 ± 3.35 min/cow. All procedures were performed aseptically. The tyloxapol dose of 120 mg/kg BW was chosen based on data from guinea pigs (Fernandez et al., 1997), and a study with 6 lactating cows, 2 Jerseys and 4 Holsteins, that received either 0 or 500 mg of tyloxapol/kg of BW with the 500 mg/kg divided into 1 (500 mg/kg), 2 (250 mg/kg), or 4 doses (125 mg/kg) spaced every 2 d (Wrenn et al., 1971). The authors reported that cows receiving the 2 doses of 250 mg/kg or a single 500 mg/kg developed brisket edema.

Blood was sampled immediately before the administration of tyloxapol, and at 10, 20, 40, 60, 120, 180, 240, 480, and 720 min later. At each sampling, 20 mL of blood was aspirated with a syringe and discarded, and then another 20 mL of blood was aspirated as the specimen used for subsequent assays. Blood was immediately transferred to one 10-mL tube containing no anticlotting agent for serum separation (Vacutainer, Becton Dickson, Franklin Lakes, NJ). Tubes were placed in ice and centrifuged within 1 h of collection at 2,000 × *g* for 20 min at room temperature for serum separation. Serum samples were aliquoted into multiple vials and frozen at −20°C until analyses. Concentrations of triacylglycerol (Stanbio Triglycerides LiquiColor Procedure No. 2100, Stanbio Laboratory, Boerne, TX) and total cholesterol (Stanbio Cholesterol LiquiColor Procedure No. 1010, Stanbio Laboratory) were analyzed using colorimetric enzymatic assays. Intra- and interassay coefficients of variation were, respectively, 3.7% and 6.4% for triacylglycerol and 1.4 and 4.8% for total cholesterol. Serum concentrations of cholesterol in VLDL particles were approximated using the equation: VLDL cholesterol = triacylglycerol/5 as reported previously (Friedewald et al., 1972). Rectal temperature and respiration and heart rates were measured at 0, 2, 5, 10, 15, 20, and 30 min relative to tyloxapol infusion. Cows with a heart rate >80 beats/min were considered to have tachycardia. Additional evaluations were recorded at 0, 2, 5, 10, 15, 20, and 30 min and continuously every 30 min for the first 720 min after tyloxapol infusion which included signs related to potential anaphylaxis such as frothy salivation, muzzle twitching, eyes twitching, muscle twitching, nystagmus, hyperexcitement, and staggering gait. Because none of the cows showed any of the clinical signs evaluated after 30 min, all data reported herein refer to the first 30 min of observation after tyloxapol infusion.

The second component of the study was to determine if the exposure to tyloxapol was associated with negative carry-over impacts on gestation length, calf birth weight, and risk of clinical diseases in early-lactation cows. For that, a companion group of 76 cows that underwent the same feed-restriction protocol but did not receive tyloxapol were used to compare with the 33 cows that received tyloxapol. The sample size was a convenience size based on the cows exposed to tyloxapol and the cohort of cows that were managed under the exact same conditions, but did not receive tyloxapol. Cows were observed daily until 21 d postpartum and abortion (gestation length <260 d and dead calf), retained placenta, metritis, mastitis, displaced abomasum, lameness, and pneumonia were recorded. Details of how these diseases were diagnosed have been described elsewhere (Bollatti et al., 2020). Morbidity included the diagnosis of at least one of the described clinical diseases.

Cow was the experimental unit in the study. For the longitudinal study, continuous data were analyzed by mixed-effects models using the MIXED procedure of SAS (ver. 9.4, SAS/STAT, SAS Institute Inc., Cary, NC). The statistical models included the fixed effects of time relative to tyloxapol infusion, the BCS on the day of study enrollment, twin birth (no vs. yes), cohort (1 to 6), and the linear effect of the response measured immediately before infusion of tyloxapol, and the random effect of cow nested within cohort. The Kenward-Roger method was used to approximate the denominator degrees of freedom to compute the *F* tests. For categorical data, descriptive statistics was used to plot histograms for the frequency of events at each time point.

For the prospective cohort study portion of this manuscript, gestation length and calf birth weight were analyzed by models that included the fixed effects of tyloxapol exposure (without vs. with tyloxapol infusion), the BCS at enrollment, and twin birth. Binary data such as the risk of individual diseases or morbidity were analyzed by generalized linear models using logistic regression with the GLIMMIX procedure of SAS fitting a binary distribution. The models included the fixed effects of exposure to tyloxapol, BCS at the time of enrollment, and twin birth. Data with fewer than 5 cases per group were analyzed by exact logistic regression using the LOGISTIC procedure of SAS and the model included the fixed effects of exposure to tyloxapol, BCS at the time of enrollment, and twin birth. The adjusted odds ratio and the respective 95% confidence intervals were computed. Continuous data are presented as least squares means and standard error of the mean, whereas categorical data are presented as frequencies with the associated adjusted odds ratio and 95% confidence interval.

Evidence of statistical significance against the null hypothesis was considered at *P* ≤ 0.05, and tendency was considered at 0.05 <*P* ≤ 0.10.

All 33 cows contributed data for concentrations of metabolites in serum and vital signs or clinical signs after infusion of tyloxapol, whereas 109 cows contributed data for gestation length, calf birth weight, risk of clinical diseases, and survival in the first 21 d postpartum. Concentrations of triacylglycerol in serum increased (*P* < 0.001) progressively from 15.6 to 40.7 mg/dL in the first 720 min relative to infusion of tyloxapol (Figure 1A). Similarly, concentrations of VLDL cholesterol and total cholesterol in serum increased (*P* < 0.001) progressively from 3.12 to 8.24 mg/dL and 120 to 138 mg/dL, respectively, in the first 720 min relative to infusion of tyloxapol (Figure 1B and 1C). Furthermore, rectal temperature increased (*P* < 0.001) from 38.3 to 38.5°C after infusion of tyloxapol, although it remained within the normothermia for dairy cows (Figure 1D). Respiration and heart rates increased (*P* = 0.01), reaching a plateau at 10 min, after which they both declined and returned to the preinfusion values by 30 min (Figures 1E and 1F). Tyloxapol induced tachycardia in 66.7% (n = 22; Figure 2A), frothy salivation in 39.4% (n = 13; Figure 2B), muzzle twitching in 15.2% (n = 5; Figure 2C), eyes twitching in 12.1% (n = 4: Figure 2D), muscle twitching in 48.5% (n = 16; Figure 2E), nystagmus in 6.1% (n = 2; Figure 2F), and signs of hyperexcitement in 18.2% (n = 6; Figure 2G), and staggering gait in 18.2% (n = 6; Figure 2H) of the cows; however, none of the cows had submandibular or generalized edema as previously reported when cows received tyloxapol in doses larger than applied in the present study (Wrenn et al., 1971). Although tyloxapol induced anaphylaxis in 12.1% (n = 4) of the cows, all signs were transient, and cows returned to normal behavior after 20 min of infusion. Abortion, gestation length, and birth weight of singleton calves were not associated with the infusion of tyloxapol (Table 1); however, in cows that carried twins, infusion of tyloxapol was associated (*P* = 0.04) with increased calf birth weight compared with cows that did not receive tyloxapol (Table 1). Infusion of tyloxapol was not associated with the risk of clinical diseases or survival in the first 21 d postpartum (Table 1).

**Figure 1:**
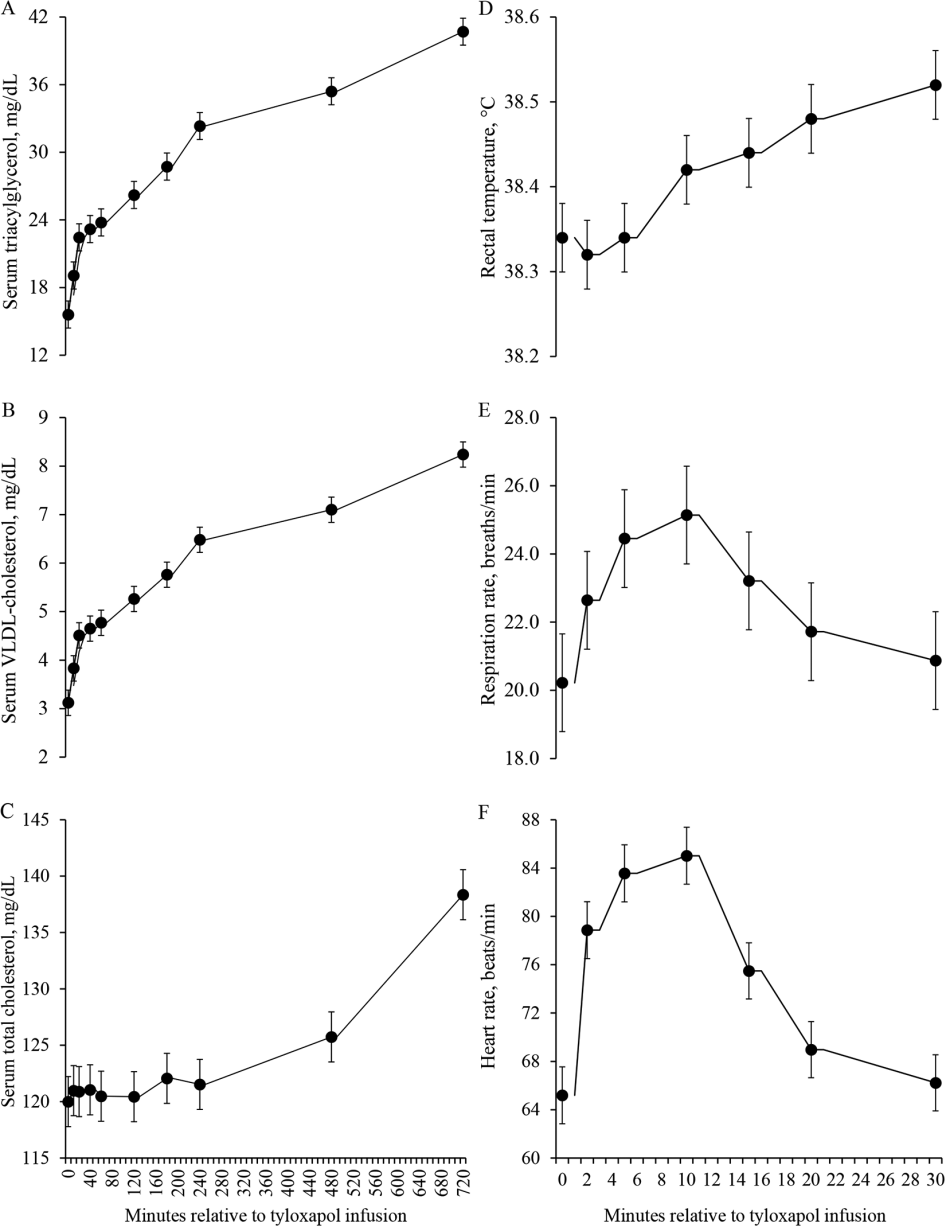
Concentrations of triacylglycerol (A), very-low-density lipoprotein (VLDL) cholesterol (B), and total cholesterol (C) in serum, and rectal temperature (D), respiration rate (E), and heart rate (F) relative to an i.v. administration of tyloxapol in 33 pregnant dry Holstein cows. Panel A: effect of time (*P* < 0.001). Panel B: effect of time (*P* < 0.001). Panel C: effect of time (*P* < 0.001). Panel D: effect of time (*P* < 0.001). Panel E: effect of time (*P* = 0.01). Panel F: effect of time (*P* < 0.001). Values represent LSM and error bars depict SEM.

**Figure 2:**
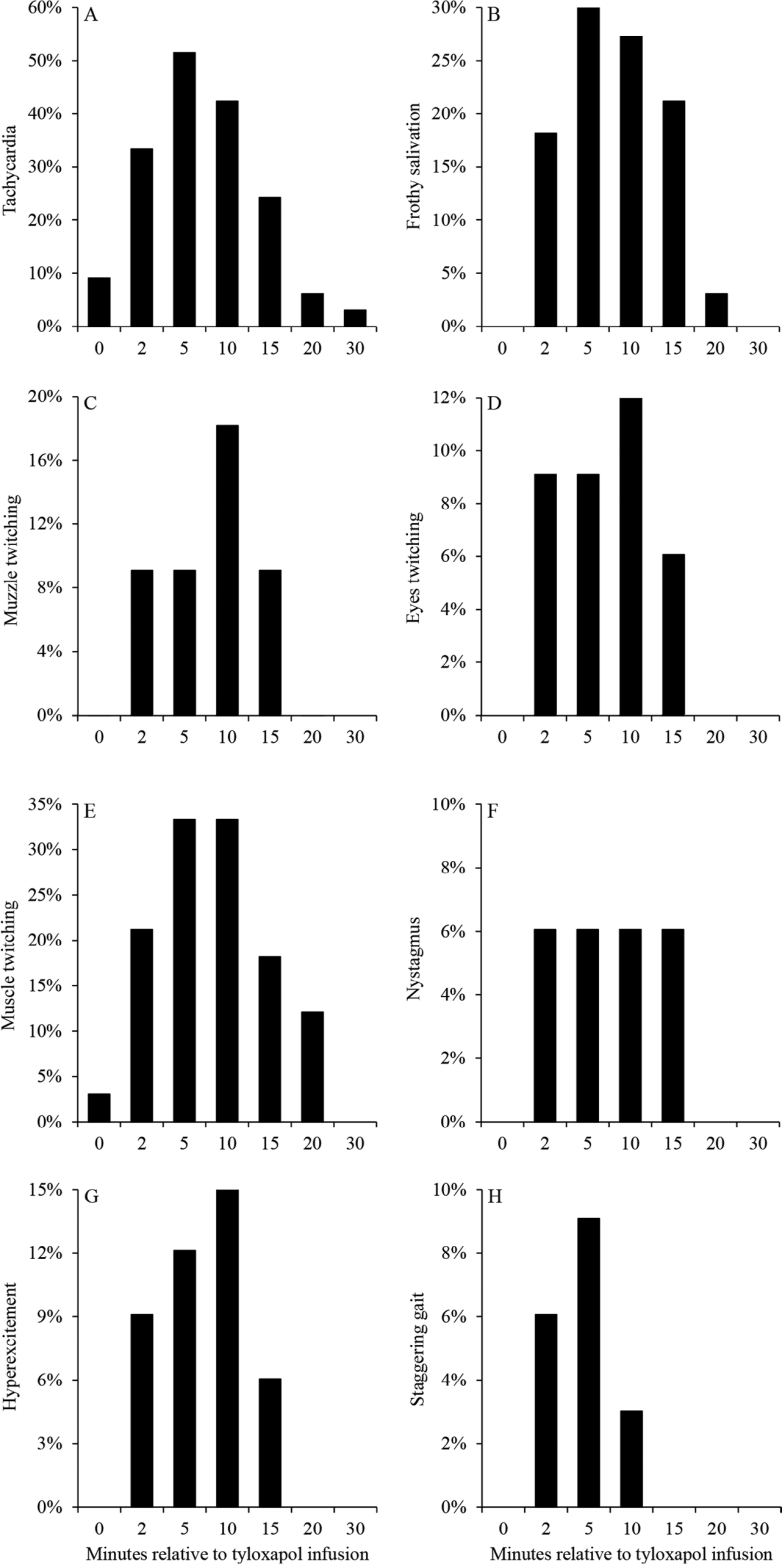
Histograms of clinical signs including tachycardia (A), frothy salivation (B), twitching of muzzle (C), eyes twitching (D), muscle twitching (E), nystagmus (F), hyperexcitement (G), and staggering gait (H) relative to an i.v. administration of 120 mg of tyloxapol per kg of BW in 33 pregnant dry Holstein cows.

**Table 1:** Associations between tyloxapol infusion and gestation length, calf birth weight, and health of dairy cows in the first 21 d postpartum (LSM ± SEM or proportions)

Item	Group^1^		AOR^2^ (95% CI)	*P*-value
	Without tyloxapol	With tyloxapol		
Cows, n	76	33	—	—
Gestation length, d	273.0 ± 1.1	272.9 ± 1.2	—	0.95
Calf birth weight, kg				
Singleton	40.5 ± 0.7	38.5 ± 1.1	—	0.13
Twin^3^	56.9 ± 2.7	65.4 ± 3.0	—	0.04
Abortion, %	1.3	6.1	1.61 (0.08–31.5)	0.75
Risk of disease, %				
Retained placenta^4^	0	6.1	—	0.95
Metritis	19.7	18.2	1.01 (0.32–3.13)	0.99
Mastitis	2.6	3.0	0.61 (0.03–11.8)	0.74
Displaced abomasum^4^	2.6	0	—	0.96
Lameness	5.3	3.0	0.16 (0.01–2.89)	0.21
Pneumonia^4^	1.3	0	—	0.95
Morbidity	32.9	27.3	0.68 (0.25–1.81)	0.44
Left herd, %	9.2	9.1	0.77 (0.13–4.53)	0.77

1 Cows underwent a period of feed restriction from d 6 to 13, and a contemporary group of 76 cows did not receive any tyloxapol, whereas 33 cows were treated with a single i.v. infusion of 120 mg of tyloxapol/kg of BW at 234 d of gestation.

2 AOR = adjusted odds ratio.

3 Five and four cows delivered twin calves in the groups without tyloxapol and with tyloxapol infusion, respectively.

4 Analyzed by the exact logistic regression because of a low frequency of events.

Infusion of tyloxapol induced progressive hypertriglyceridemia and hypercholesterolemia in feed-restricted cows, and such responses resemble those shown previously in rats (Lombardi et al., 1968) and goats (Schultz and Esdale, 1971). Cows had a substantial accumulation of lipids in serum, and the extent of response likely extended for hours or days after the last sampling time at 720 min after tyloxapol infusion (Wrenn et al., 1971). The increased accumulation of lipids in blood suggests reduced hydrolysis by lipoprotein lipase; however, the rate of increase seems slower than observed in guinea pigs (Fernandez et al., 1997), probably because ruminant hepatocytes are known to have reduced de novo lipogenesis (Hanson and Ballard, 1968) and ability to export triacylglycerol as VLDL (Emery et al., 1992). Schultz and Esdale (1971) kept 2 Saanen goats off feed for 72 h and i.v. infused a solution containing 20% Triton WR-1339 at 200 mg/kg BW. Starting at 48 h, blood was collected sequentially for 216 h and the authors showed that concentrations of triacylglycerol in plasma progressively increased until 72 h postinfusion, whereas those of phospholipids and total cholesterol increased until 96 h after infusion. The progressive increase in concentrations of lipids in blood suggests that quantification of triacylglycerol-rich lipoprotein should be longer than the 12 h used in the present study. It is likely that concentrations of triacylglycerol and cholesterol increased further beyond 720 min. Because cholesterol is carried primarily by high-density lipoproteins in ruminants (Bauchart, 1993), the changes in concentration over time after tyloxapol infusion might reflect hepatic secretion, but also a possible reduction in tissue uptake of lipoprotein cholesterol. It would be interesting to combine the use of tyloxapol to reduce lipolysis in lipoproteins with infusion of carbon-labeled lipids to evaluate the rate of disappearance from blood.

Although tyloxapol caused transient clinical signs in cows, those were of short duration, lasting 20 min. Most cows experienced tachycardia, which suggests some degree of hypotension that was compensated for an increased heart rate. Although unlikely, one could argue that a drop in blood pressure might be because of increments in blood lipids and reduced force of blood moving through the blood vessels. Changes in heart rate were observed shortly after infusion, whereas changes in blood lipid concentrations took longer to be observed. As opposed to Wrenn et al. (1971), no lower body edema was observed in any of the cows treated, likely because the dose used was smaller than those used by those authors. In goats fed ad libitum, infusion of 200 or 400 mg of Triton WR-1339/kg of BW did not affect intake or milk production, or cause edematous swellings on the body (Schultz and Esdale, 1971). In low-producing dairy cows, infusion of 500 mg/kg Triton WR-1339 reduced intake and milk yield (Wrenn et al., 1971). As expected, responses to tyloxapol are dose-dependent and limiting the amount infused is important for safety of cows during studies of dynamics of blood lipids. Although 4 of the 33 cows treated showed signs of anaphylaxis, all 4 cows recovered within the first 20 min of infusion without the use of any therapy. Also, induction of hyperlipidemia did not seem to affect gestation length or health of cows in the subsequent lactation.

The approach used in this study was based on extrapolating the literature from other species (Schultz and Esdale, 1971; Fernandez et al., 1997) and from dairy cattle (Wrenn et al., 1971) to select the dose to be used. It is possible that a smaller dose might be adequate to study hyperlipidemia in dairy cows and perhaps resulting in fewer side effects. In laboratory animals, tyloxapol associates with triacylglycerols in lipoprotein particles in blood plasma resulting in reduced action by lipoprotein lipase, thus limiting hydrolysis of components of lipoproteins and subsequent uptake of these lipids by the extra hepatic tissues. These action allow for estimation of the rate of entry of triacylglycerols into the plasma pool (Otway and Robinson, 1967; Fernandez et al., 1997). Such properties make tyloxapol attractive to study the rate of entry of lipids into the circulation so long as it does not result in toxic effects.

Tyloxapol induced hyperlipidemia based on the progressive increases in concentrations of triacylglycerol, VLDL cholesterol, or total cholesterol in serum. Tyloxapol induced transient reactions that were limited to the first 20 min after treatment, but the dose of 120 mg/kg BW was not associated with subsequent negative impacts on gestation length or health of dairy cows in the first 21 d postpartum. It is noteworthy to mention that this model was established in feed-restricted dry Holstein cows at 8 mo of gestation, and the transient reactions after tyloxapol infusion might potentially have side effects in pregnant cows at earlier stages of gestation. Also, the dose of tyloxapol and implications of this model for lactating cows might differ and warrant further investigation. The application of this model would be useful to study intermediary lipid metabolism in dry third-trimester pregnant dairy cows.

## References

[bib1] Arshad U., Husnain A., Poindexter M.B., Zimpel R., Nelson C.D., Santos J.E.P. (2023). Rumen-protected choline reduces hepatic lipidosis by increasing hepatic triacylglycerol-rich lipoprotein secretion in dairy cows. J. Dairy Sci..

[bib2] Bauchart D. (1993). Lipid absorption and transport in ruminants. J. Dairy Sci..

[bib3] Bell A.W., Slepetis R., Ehrhardt U.A. (1995). Growth and accretion of energy and protein in the gravid uterus during late pregnancy in Holstein cows. J. Dairy Sci..

[bib4] Bobe G., Young J.W., Beitz D.C. (2004). Invited review: Pathology, etiology, prevention, and treatment of fatty liver in dairy cows. J. Dairy Sci..

[bib5] Bollatti J.M., Zenobi M.G., Artusso N.A., Lopez A.M., Nelson C.D., Barton B.A., Staples C.R., Santos J.E.P. (2020). Effects of rumen-protected choline on the inflammatory and metabolic status and health of dairy cows during the transition period. J. Dairy Sci..

[bib6] Emery R.S., Liesman J.S., Herdt T.H. (1992). Metabolism of long chain fatty acids by ruminant liver. J. Nutr..

[bib7] Fernandez M.L., Vergara-Jimenez M., Conde K., Behr T., Abdel-Fattah G. (1997). Regulation of apolipoprotein B-containing lipoproteins by dietary soluble fiber in guinea pigs. Am. J. Clin. Nutr..

[bib8] Friedewald W.T., Levy R.I., Fredrickson D.S. (1972). Estimation of the concentration of low-density lipoprotein cholesterol in plasma, without use of the preparative ultracentrifuge. Clin. Chem..

[bib9] Hanson R.W., Ballard F.J. (1968). The metabolic fate of the products of citrate cleavage. Adenosine triphosphate citrate lyase and nicotinamide–adenine dinucleotide phosphate-linked malate dehydrogenase in foetal and adult liver from ruminants and non-ruminants. Biochem. J..

[bib10] Jorritsma R., Jorritsma H., Schukken Y.H., Bartlett P.C., Wensing T., Wentink G.H. (2001). Prevalence and indicators of postpartum fatty infiltration of the liver in nine commercial dairy herds in the Netherlands. Livest. Prod. Sci..

[bib11] Lombardi B., Pani P., Schlunk F.F. (1968). Choline-deficiency fatty liver: Impaired release of hepatic triglycerides. J. Lipid Res..

[bib12] NASEM (National Academies of Sciences, Engineering, and Medicine) (2021).

[bib13] Otway S., Robinson D.S. (1967). A non-ionic detergent (Triton WR1339) to determine rates of triglyceride entry into the circulation of the rat under different physiological conditions. J. Physiol..

[bib14] Rinella M.E., Elias M.S., Smolak R.R., Fu T., Borensztajn J., Green R.M. (2008). Mechanisms of hepatic steatosis in mice fed a lipogenic methionine choline-deficient diet. J. Lipid Res..

[bib15] Schotz M.C., Scanu A., Page I.H. (1957). Effect of Triton on lipoprotein lipase of rat plasma. Am. J. Physiol..

[bib16] Schultz L.H., Esdale W.J. (1971). Triton-induced hyperlipemia in goats under various physiological conditions. J. Dairy Sci..

[bib17] Wrenn T.R., Weyant J.R., Miller R.W., Bitman J. (1971). Alterations in blood and milk lipids produced by administration of Triton WR-1339. J. Dairy Sci..

